# Quantifying Deformation and Migration Properties of U87 Glioma Cells Using Dielectrophoretic Forces

**DOI:** 10.3390/bios12110946

**Published:** 2022-10-31

**Authors:** Meltem Elitas, Monsur Islam, Jan G. Korvink, Esra Sengul, Pouya Sharbati, Beyzanur Ozogul, Sumeyra Vural Kaymaz

**Affiliations:** 1 Faculty of Engineering and Natural Sciences, Sabanci University, Istanbul 34956, Turkey; 2 Institute for Microstructure Technology, Karlsruhe Institute of Technology, 76344 Karlsruhe, Germany; 3 Department of Physiology Anatomy and Genetics, University of Oxford, Oxford OX1 3PT, UK

**Keywords:** dielectrophoresis, deformability, migration, displacement, glioma, GBM

## Abstract

Glioblastoma multiforme is one of the most aggressive malignant primary brain tumors. To design effective treatment strategies, we need to better understand the behavior of glioma cells while maintaining their genetic and phenotypic stability. Here, we investigated the deformation and migration profile of U87 Glioma cells under the influence of dielectrophoretic forces. We fabricated a gold microelectrode array within a microfluidic channel and applied sinusoidal wave AC potential at 3 V_pp_, ranging from 30 kHz to 10 MHz frequencies, to generate DEP forces. We followed the dielectrophoretic movement and deformation changes of 100 glioma cells at each frequency. We observed that the mean dielectrophoretic displacements of glioma cells were significantly different at varying frequencies with the maximum and minimum traveling distances of 13.22 µm and 1.37 µm, respectively. The dielectrophoretic deformation indexes of U87 glioma cells altered between 0.027–0.040. It was 0.036 in the absence of dielectrophoretic forces. This approach presents a rapid, robust, and sensitive characterization method for quantifying membrane deformation of glioma cells to determine the state of the cells or efficacy of administrated drugs.

## 1. Introduction

Glioblastoma multiforme (GBM, Grade IV glioma) is one of the most invasive and aggressive types of malignant primary brain tumors in humans. Although the occurrence of brain tumors is relatively low among other types of cancers, the mortality rate of GBM is still the highest [1]. In GBM, complex, dynamic, and heterogeneous microenvironments of glioma cells, sizes, and locations of primary tumors in the critical areas of the brain, surrounding the brain tissue by the brain-blood barrier and the skull challenge the administration of the treatment strategies [2,3]. Surgery, radiation therapy, and chemotherapy have limited outcomes, with a 15-month median survival rate [4,5]. Although its spread is diffusely infiltrative, metastases are very rare, and brain metastases development can cause death in up to 50% cases [6,7,8,9]. Therefore, mechanistically understanding the behavior of glioma cells is critical to designing preventives and effective treatment strategies for improved survival of patients [6,9].

Over the years, several studies have reported that differences in the mechanical properties of cells, particularly the alterations in cell stiffness, can be used to detect malignant transformation of cells with similar shapes or to investigate their mechanisms of metastatic competency [10,11,12,13,14,15]. Changes in morphology, motility, adhesion, deformation, and invasion characteristics of cells are tightly linked to the dynamic reorganization of their cytoskeleton [10,12,16,17]. Several biomechanical assays have been developed to evaluate and sense the deformation changes in tumor cells. In the literature, most of the cell-deformation investigations have been performed using micropipette aspiration [18,19], fluorescence microscopy [16], atomic force microscopy [17,19,20,21], single-cell force spectroscopy [17,22], ektacytometer [23], optical tweezers [13,24], laser traps [25], and shear-based microfluidic assays [26,27]. Although these tools are very effective for quantitative single-cell analysis, still they might alter genetic and phenotypic properties of the cells. Besides, they are often limited by expensive and bulky experimental systems.

Here, we used dielectrophoresis (DEP) to quantify the deformation and motility heterogeneity of the U87 glioma cell line, which is one of the most aggressive brain cancer cell lines [1,2,3,4,5,6,7,8,9,10]. Variations in the deformation behavior of cells can be considered as a marker for the detection of phenotypic differences [20,28,29]. DEP can be defined as the mobility of electrically polarizable cells generated by non-uniform electric fields [30,31]. Dielectrophoretic forces are the gentlest forces that do not influence cell viability or cell morphology as shear forces [31] or magnetic forces [32]. Notably, the dielectrophoretic forces influencing the cells rely on the polarizability of the cells, which is directly linked to the properties of their cell membrane. Precisely quantifying membrane features of glioma cells might identify indicators to reveal their phenotypical differences, such as morphology, deformation, migration pattern, and motility, in the complex microenvironments [10,33]. Moreover, in DEP applications, the large footprint of the interdigitated electrodes allows simultaneous measurement of dielectrophoretic deformation of several cells without any physical interaction. Therefore, DEP might be a rapid, robust, and sensitive characterization method for quantifying membrane deformation of cancer cells to determine the state of the cells or the efficacy of treatment.

Recent progress in the study of the dielectrophoretic cell-deformation investigations focused on the shape transformation of red blood cells and white blood cells to investigate the activation of their membrane-bound proteins or repairment mechanisms to deformations due to chemical and physical changes that interfere with their cytoskeleton-bilayer [34,35,36,37,38,39]. Among these studies, Gass and co-workers investigated the mechanical properties of human red blood cells and their shape transformations due to the local deformations induced by high-frequency electric field [39]. Menachery et al. used an array of microelectrodes to apply dielectrophoresis for the characterization of dendritic cell deformation upon maturation [28]. Chan et al. identified electrical parameters that show cell shrinkage and blebbing on a body of MDA-MB-231 breast cancer cells [29]. Guido’s group used dielectrophoresis to investigate deformability differences between cancerous (MCF-7) and noncancerous (MCF-10F) breast cancer cell lines [40]. Chen and co-workers presented the electrodeformation differences between two cervical cancer cell lines, SiHa and ME180, and they compared their electrodeformation results with the results of the conventional micropipette aspiration technique [10]. Huang and co-workers combined microfluidics, dielectrophoresis, and optics to measure multiple cellular biophysical properties of cancer cells and leukocytes [41,42]. In this study, we developed a gold microelectrode array and applied sinusoidal wave AC potential at 3 V_pp,_ ranging from 30 kHz to 10 MHz frequencies, to generate DEP forces to measure dielectrophoretic movement and deformation changes in glioma cells.

## 2. Materials and Methods

### 2.1. Theory

In this study, the dielectrophoretic forces $\left(F_{DEP}\right)$ induced the translational movement and/or deformation of glioma cells were described by Herbert Pohl in the 1950s, Equation (1), [30,31,43].

(1) $$F_{DEP}=2\pi \varepsilon_{m}r^{3}Re\left[f_{CM}\left(\omega \right)\right]\nabla \left|E_{rms}^{2}\right|,$$

**Figure 1:**
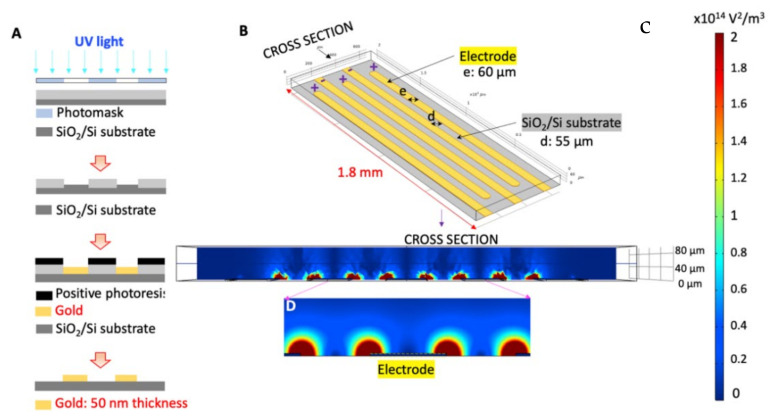
DEP chip (**A**) Fabrication process of the gold electrode array, (**B**) Organization of the 65 pairs of electrodes in the microfluidic chip, (**C**) Distribution of the electric field gradient (V^2^/m^3^) at the cross-section view of the device using COMSOL Multiphysics simulation [45].

**Figure 2:**
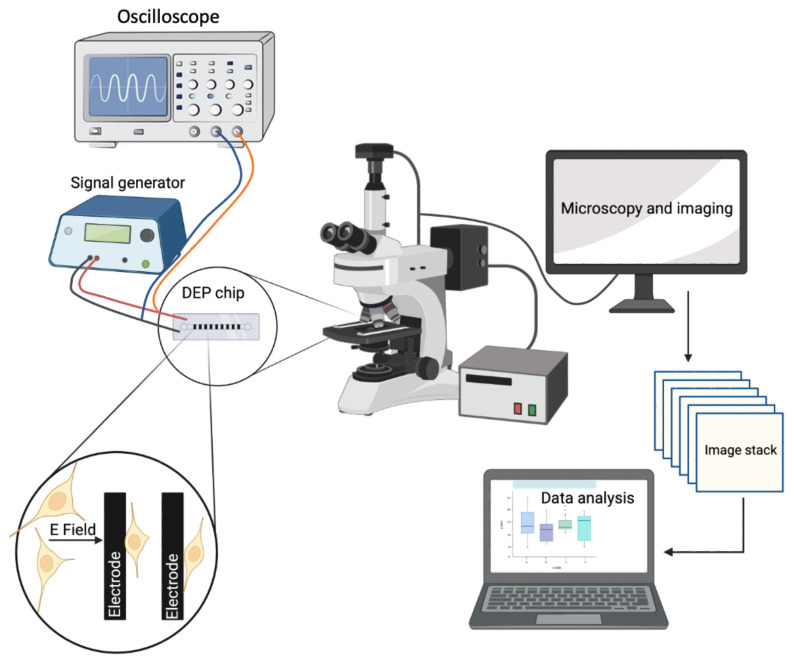
Schematic of the experimental setup including an oscilloscope, signal generator, DEP chip, table-top upright microscope, and a computer.

In Equation (1), $\varepsilon_{m}$ is the permittivity of the suspending medium, *r* is the radius of a cell, *d* is the membrane thickness of a cell, RefCMω is the real part of the Clausius-Mossotti factor, *E* is the applied electric field, ω is the angular frequency (ω=2πf), where *f* is the applied frequency. The Clausius-Mossotti factor $f_{CM}\left(\omega \right)$ is defined as given in Equation (2). The *Re*$\left[f_{CM}\left(\omega \right)\right]$ is bounded by the values of $-0.5\leq Re\left[f_{CM}\left(\omega \right)\right]\leq 1.0$ and it can define either positive dielectrophoretic (pDEP) forces or negative dielectrophoretic (nDEP) forces are generated in the microelectrode array [10,11,33,34]. When the *Re*$[f_{CM}\left(\omega \right)]>0$, the cells become more polarizable than their suspending medium, they move towards the strongest electric field regions (The edges of the gold electrodes, Figure 1 and Figure 2). By contrast, when the *Re*$[f_{CM}\left(\omega \right)]<0$, the nDEP forces are created in the microfluidic device, these forces repel the less polarizable cells in the more polarizable medium towards the weakest electric field regions. The crossover frequency denotes the frequency at which there are almost no forces acting on the cells [10,11].

(2) $$f_{CM}\left(\omega \right)=\frac{\varepsilon_{eff}^{*}-\varepsilon_{m}^{*}}{\varepsilon_{eff}^{*}+2\varepsilon_{m}^{*}},$$

(3) $$\varepsilon_{m}^{*}=\varepsilon_{m}-j\frac{\sigma_{m}}{\omega },$$

where $\varepsilon_{eff}^{*}$ indicates the complex permittivity of the cell, $\varepsilon_{m}^{*}$ indicates the complex permittivity of the DEP medium (3). $\sigma_{m}$ is the conductivity value of the medium. *j* is $\sqrt{-1}$, $\varepsilon_{eff}^{*}$ indicates complex permittivity of a cell (4), $\varepsilon_{int}^{*}$ and $\varepsilon_{mem}^{*}$ represents complex permittivity of cytoplasm and complex permittivity of membrane, respectively.

(4) $$\varepsilon_{eff}^{*}=\varepsilon_{mem}^{*}\frac{{\left(\frac{r}{r\;-\;d}\right)}^{3}+2\frac{\varepsilon_{int}^{*}\;-\;\varepsilon_{mem}^{*}}{\varepsilon_{int}^{*}\;+\;2\varepsilon_{mem}^{*}}}{{\left(\frac{r}{r\;-\;d}\right)}^{3}-\frac{\varepsilon_{int}^{*}\;-\;\varepsilon_{mem}^{*}}{\varepsilon_{int}^{*}\;+\;2\varepsilon_{mem}^{*}}},$$

### 2.2. Cell Preparation

We used the U87 MG (HTB-14™) human glioma cell line, which is highly aggressive type of glioblastoma cell line [2,3]. We purchased the U87 MG (HTB-14™) glioma cells from ATCC (American Type Culture Collection, Manassas, VA, USA). U87 glioma cells were maintained in DMEM medium (Dulbecco’s modified Eagle’s medium, PAN-Biotech, Bayern, Germany) supplemented with 10% fetal bovine serum (FBS/Sigma Aldrich, St. Louis, MO, USA), and 1% Penicillin/Streptomycin (PAN-Biotech, Bayern, Germany). We cultured the cells in the 75 cm^2^ flasks (TTP, Switzerland) at 37 °C with 5% CO_2_ in the incubator (EC 160 CO_2_-Nuva, Ankara, Turkey). When the cells became confluent, we stripped them from the growth medium using trypsin solution (PAN-Biotech, Bayern, Germany). The cell population of 1 × 10^6^ cells/mL was counted using a hemocytometer (Paul Marienfeld GmbH & Co. KG, Lauda-Königshofen, Germany). Next, the low conductive DEP buffer consisting of 8.6% sucrose (BioFroxx, Hesse, Germany), 0.3% glucose (Sigma Aldrich, St. Louis, MI, USA), and 0.1% Bovine Serum Albumin (BSA, PAN-Biotech, Bayern, Germany) was prepared in deionized water. The conductivity value of the suspension was set to 20 µS/cm verified by measuring a conductivity meter (CORNING, 311 Conductivity, Merck KGaA, Darmstadt, Germany) [43,44]. Afterwards, glioma cells were spun down at 3000 rpm (Hettich EBA 20 Centrifuge, Merck KGaA, Darmstadt, Germany) for 5 min to wash the cells using the DEP buffer twice. The viability of cells was determined in the low conductive DEP buffer by incubating the cells in the DEP buffer for 30 min using Trypan Blue dye (Thermo Fisher Scientific, Waltham, MA, USA). In the absence of electric field exposure, we determined the survival of glioma cells in the DEP buffer, the viability of freshly prepared cells was 6 × 10^5^ cells/mL and 5.6 × 10^5^ cells/mL after 30 min (Unpaired *t*-test, not significant (ns), *p*-value 0.26). The use of low conductive DEP buffer reduces the electrical conductivity-induced heat, which scales with conductivity [41,45].

### 2.3. DEP Chip, Setup and Experimental Procedure

The DEP chip consists of 65 pairs of electrodes in an interdigitated organization to generate a non-uniform electric field. The gold electrodes were fabricated at 40-nm height, 1.8-mm length, and 60-µm width using the lift-off process as detailly reported by Sengul et al. [44], Figure 1A. We used a cutting plotter machine (Graphtech CE6000-40, Yokohama, Japan) to create a 100-µm thick microfluidic channel on the electrode array using a double-sided pressure-sensitive adhesive sheet (PSA). We assembled the device using a 3-mm thick polymethyl methacrylate (PMMA) layer, Figure 1B. To introduce the cells into the device, we used a CO_2_ Laser-Engraving machine (ULS Versa Laser 3.50, 10.6 µm wavelength) to make an inlet and outlet on the PMMA cover [44].

To investigate the optimal DEP conditions to manipulate U87 glioma cells in the microelectrode array, Sharbati and co-workers performed the numerical analysis using COMSOL Multiphysics 5.6 program in [45,46]. Figure 1C presents the distribution of the electric field gradient in the microfluidic channel when the AC potential at 3 V_pp_ ranging from 10 kHz to 150 kHz frequencies was applied, Figure 1 [45,46].

We performed the experiments using the experimental setup shown in Figure 2. We have a signal generator (Model: GFG-8216A, GW Instek, New Taipei City, Taiwan) to apply sinusoidal wave AC potential at peak-to-peak voltage of 3 V_pp_ at the frequency range of 30 kHz to 10 MHz. We observed the applied signal using an oscilloscope (Part Number: 54622D, Agilent Technologies, Santa Clara, CA, USA). We acquired the images of the cells using an upright microscope (Model: Nikon ME600 Eclipse, Nikon Instruments Inc., Melville, NY, USA). We used a computer to save and analyze the acquired images (HP).

**Figure 3:**
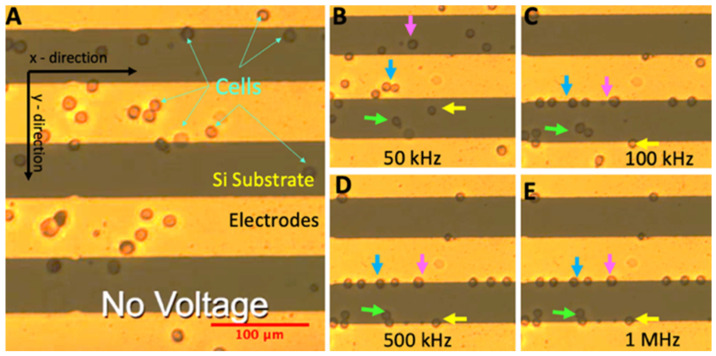
U87 glioma cells in the electrode array. Movement of glioma cells when the electric field was (**A**) off and when 3 V_pp_ at (**B**) 50 kHz, (**C**) 100 kHz, (**D**) 500 kHz, and (**E**) 1 MHz frequencies were applied. Arrows show the movement of glioma cells. The scale bar is 100 µm.

Prior to loading the glioma cells into DEP device, we sterilized the device using 70% isopropyl alcohol and rinsed the device with deionized water and the DEP buffer, respectively [43,44]. In addition, we used the DEP buffer to remove bubbles from the electrode array. The experiment was initiated by loading 40 μL of cell suspension (6 × 10^5^ cells/mL) in the microelectrode array using a 200-µL pipette. DEP experiments were performed without fluid flow (there was no drag force in the device). The movement of the glioma cells was generated by dielectrophoretic forces in the DEP device. When there was no cell flow in the electrode array (due to pressure differences in the inlet and outlet ports), we applied 3 V_pp_, 30 kHz signal and acquired the images of the cells at 5 frames/second. We performed the experiments in 30 min. Figure 3A illustrates the acquired micrograph of the electrode array with glioma cells in the absence of an electric field, Figure 3. Movement of the glioma cells was demonstrated in Figure 3B,E when 3 V_pp_, 50 kHz (Figure 3B), 100 kHz (Figure 3C), 500 kHz (Figure 3D), and 1 MHz (Figure 3E) were applied. The experiments were performed at least in triplicate.

Next, we used ImageJ software (NIH, Bethesda, MD, USA) to manually measure area, perimeter, and coordinates of the cells in the acquired image sequences when glioma cells were exposed to 3 V_pp_ and 30 kHz, 40 kHz, 50 kHz, 60 kHz, 70 kHz, 80 kHz, 90 kHz, 100 kHz, 500 kHz, 1 MHz, 2 MHz, 5 MHz, and 10 MHz frequencies. We presented the data using OriginPro 2021b (OriginLab Corporation, Northampton, MA, USA) and GraphPad Prism programs. We performed ordinary one-way ANOVA Tukey’s multiple comparison test and Student’s unpaired *t*-test (two-tailed using the GraphPad Prism software. The *p*-value was considered significant: * *p* ≤ 0.05, ** *p* ≤ 0.01, *** *p* ≤ 0.001, **** *p* ≤ 0.0001.

## 3. Results

**Figure 4:**
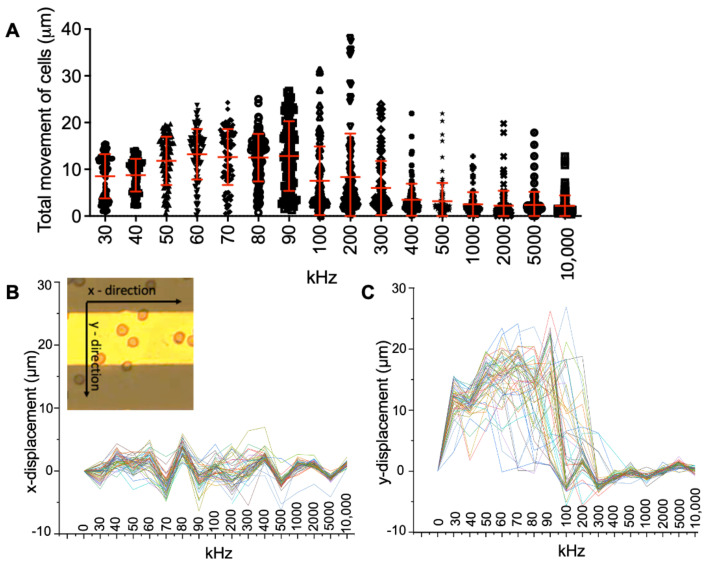
Dielectrophoretic displacement of U87 glioma cell population. (**A**) Distribution of total displacement, movement of glioma cells along (**B**) *x*-axis, and (**C**) *y*-axis when the cells were exposed to 3 V_pp_ and 30 kHz–10 MHz frequencies. The inset shows the directions in the electrode array. The scale bar is 100 µm. 100 glioma cells were analyzed at each frequency, the red bars show means ± standard deviations.

Our objective was to use DEP for the deformation and migration characterization of U87 glioma cells. Dielectrophoretic polarizability can provide intrinsic properties of a cell related to permittivity and conductivity of a cell membrane and cytoplasm, Equations (1)–(4). Here, we obtained deformation indexes [47,48,49,50] and migration distances of glioma cells under the influence of DEP forces. We exposed glioma cells to the non-uniform electric field by applying sinusoidal wave AC potential 3 V_pp_ voltage at 30 kHz, 40 kHz, 50 kHz, 60 kHz, 70 kHz, 80 kHz, 90 kHz, 100 kHz, 200 kHz, 300 kHz, 400 kHz, 500 kHz, 1 MHz, 2 MHz, 5 MHz, 10 MHz frequencies. Next, we measured the coordinates of glioma cells in each frequency, Figure 3 and Figure 4. Afterwards, we determined the displacements of cells in the electrode array using the following Equation (5).

(5) $$\Delta r_{t}=\sqrt{{\left(x_{t+\Delta t}-x_{t}\right)}^{2}+{\left(y_{t+\Delta t}-y_{t}\right)}^{2}},$$

where $\Delta r_{t}$ represents displacement of a glioma cell at the measured coordinates of $x_{t}$ and $y_{t}$, and Δt denotes the time interval between two consecutive time points t. Figure 4 shows dielectrophoretic movement of 100 glioma cells at each frequency.

Figure 4A illustrates the distributions of total displacements of glioma cells for each frequency with means and standard deviations. The initial positions of the cells were normalized to zero in Figure 4B,C. We observed that U87 glioma cells exhibited crossover frequency around 100–200 kHz [35]. The heterogeneity of U87 glioma cells was not visible upon the crossover frequency region since all the cells were strongly attracted by electrodes. However, the distribution of dielectrophoretic displacement was wider around crossover frequency (90–300 kHz). The distribution of the glioma distances was not uniform at 200 kHz, Figure 4A–C displays the movement of 100 glioma cells according to their dielectrophoretic responses along the *x*-axis and *y*-axis (inset in Figure 4B), respectively. Next, we calculated the dielectrophoretic deformation indexes of U87 glioma cells using Equation (6), where *π* is 3.14 [47,48,49,50].

(6) $$DDI=1-\frac{2\sqrt{\pi \;\mathrm{Area}}}{\mathrm{Perimeter}},$$

**Figure 5:**
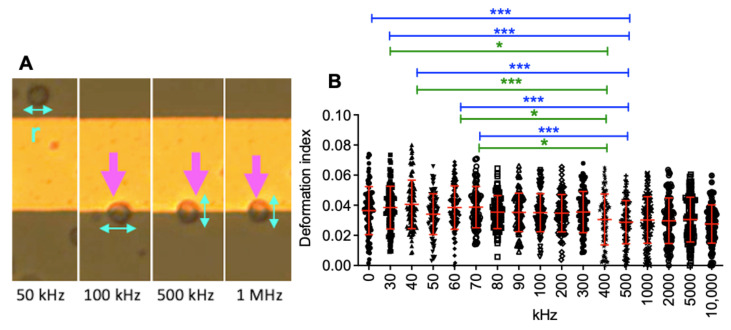
Dielectrophoretic deformation of U87 glioma cells. (**A**) Deformation of a cell at 3 V_pp_–50 kHz, 100 kHz, 500 kHz, and 1 MHz, (**B**) Deformation indexes of cells at 0 kHz–0 Vpp, and 3 V_pp_–30 kHz to 10 MHz. One-way analysis of variance test was applied. n = 100 cells, * = *p* < 0.05, *** = *p* < 0.001.

Figure 5 displays the deformation indexes of U87 glioma cells at 0 kHz–0 V_pp_ (Cells were at rest) and 3 V_pp_–30 kHz to 10 MHz frequencies. The dielectrophoretic deformation indexes of U87 glioma cells altered between 0.027–0.040, Table 1. We determined the significant differences between the deformation indexes of the glioma cells for the applied frequencies using One-way analysis of variance, Tukey’s Multiple Comparison Test. The mean dielectrophoretic deformation index of glioma cells was 0.036 ± 0.01 (mean ± standard deviation, n = 100 glioma cells) in the absence of dielectrophoretic forces. When the cells experienced strong pDEP forces at 3 V_pp_, 500 kHz–10 MHz, there was not a significant difference in the deformation indexes (~0.028 ± 0.01) contrary to 3 V_pp_, 30–500 kHz (mean ± standard deviation, n = 100 glioma cells at each frequency value).

**Figure 6:**
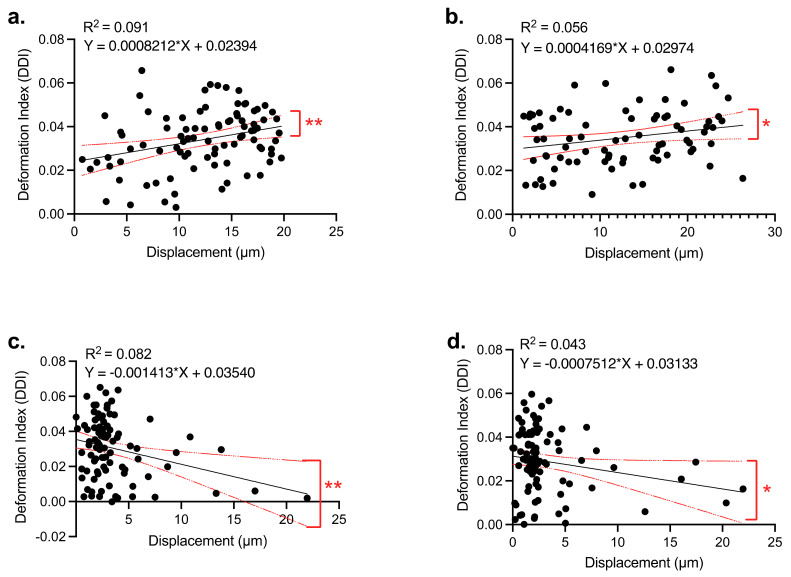
Linear regression between the deformation indexes and displacements of U87 glioma cells. When 3 V_pp_ and (**a**) 50 kHz, (**b**) 90 kHz, (**c**) 400 kHz, and (**d**) 500 kHz electric field was applied. Simple linear regression test was applied. n =100 cells at each frequency, * = *p* < 0.05, ** = *p* < 0.01.

Figure 6 presents that there is a positive linear relationship between the deformation indexes and displacement of glioma cells when the cells were exposed to nDEP forces at 50 kHz (Figure 5A, *p* = 0.0038) and 90 kHz (Figure 6b, *p* = 0.0369). Contrary, when the cells were attracted by pDEP forces at 400 kHz (Figure 6c, *p* = 0.0054) and 500 kHz (Figure 6d, *p* = 0.0473), we observed a negative linear relationship between the displacement and deformation indexes of the cells. However, we did not obtain a linear relationship between the deformation indexes and travel distances of glioma cells for the rest of the frequencies. Dielectrophoretic deformation indexes of glioma cell populations shifted from 0.04–0.045 to 0.030–0.035 when the pDEP forces were generated at the frequency range of 5 MHz to 10 MHz.

## 4. Discussion

Cellular deformability, in general, is an important characteristic that defines the physiological state of cells. In the literature, it is well documented that differences in the mechanical properties of cells correspond to the pathophysiological states of cells in several diseases [34,35,36,37,38,39,40,41,42]. Alterations in the membrane or cytosolic properties of cells might affect their ability to deform, repairment mechanisms, or influence their migration patterns [34,35,36,37,38,39]. Our DEP approach is suitable for the measurement of deformation and displacement of single cells using mostly pDEP forces. In an array of interdigitated microelectrodes, precise position and deformation measurement of single cells can be performed in a stable manner without appreciable contact with electrodes. Hundreds of single cells can be manually analyzed via ImageJ software, using an automated image analysis tool; the number of analyzed single cells can be increased. This device is not convenient for the observation of clear nDEP or crossover regions in the electrode array due to the uniform design of the electrodes. Hence, we could provide a range of 100–200 kHz frequencies for the crossover frequency as reported in [43,44,45]. This fact is one limitation of our approach, as 100 cells cannot provide the complete heterogeneity profile of the whole glioma population. However, it is one of the first studies that continuously monitored 100 glioma cells in the device for the deformation and displacement alterations by DEP forces. Mostly, tens of cells were analyzed for on-chip single-cell analysis [38,39,40,41,42]. Here, we provide a proof-of-concept by analyzing 100 glioma cells at each frequency, and we exhibited its potential in label-free deformation analysis of mammalian cells. Considering its simple fabrication, practical usage, and rapid assay time, we believe that this approach can be a powerful tool in conjunction with automated image analysis.

These experiments were conducted in an aqueous ionic fluid, and hence it is vital to consider the influence of other electrokinetic forces that might contribute to shear flow-induced stretching. In addition to DEP, the other most frequently reported phenomena are AC electro-osmotic flow and electrothermal flow [41,44,45,46,50]. With the low-medium conductivity of 20 µS/cm used in our experiments, it is unlikely that electrothermal flow is a contributing factor. Since most of the cancer cell lines have not been investigated using dielectrophoretic deformation, our objective was, therefore, to use dielectrophoresis for the deformation characterization of U87 glioma cells.

## 5. Conclusions

We fabricated a microfluidic-microelectrode array to quantify heterogeneity of U87 glioma cell line according to dielectrophoretic displacement and deformation indexes of 100 glioma cells. We applied 3 V_pp_ voltage at 30 kHz, 40 kHz, 50 kHz, 60 kHz, 70 kHz, 80 kHz, 90 kHz, 100 kHz, 200 kHz, 300 kHz, 400 kHz, 500 kHz, 1 MHz, 2 MHz, 5 MHz, 10 MHz frequencies. We showed that glioma cells exhibited the response of crossover frequency between 100–200 kHz. The mean dielectrophoretic displacements of glioma cells were significantly different at varying frequencies, with the maximum and minimum travel distances of 13.22 µm at 60 kHz and 2.18 µm at 10 MHz, respectively. Dielectrophoretic displacement of glioma cells was widely distributed around crossover frequency (90–300 kHz). There were distinct subpopulations of glioma cells at 200 kHz. However, the heterogeneity of U87 glioma cells was not visible except the crossover frequency, particularly for the pDEP frequencies, since all the cells were strongly attracted by electrodes. Deformation indexes of U87 glioma cells were measured as 0.036 in the absence of dielectrophoretic forces. When we applied 3 V_pp_ and 30 kHz to 10 MHz frequencies, we monitored that the mean deformation indexes of U87 glioma cells varied between 0.027–0.040.

Our future work will consider the optimization of the electrode array to increase precision and accuracy of measurements. Besides, we will focus on underlying the biophysical differences in the subpopulations of U87 glioma cells, which showed differences in dielectrophoretic motility and deformation indexes, using immunostaining of cytoskeletal proteins.

## Figures and Tables

**Table 1:** Dielectrophoretic displacements and deformation indexes of U87 glioma cells. Displacement and deformation values were represented by means ± STD, n = 100 cells.

Frequency (kHz)	Displacement (µm)	Deformation Indexes
30	8.51 ± 4.73	0.036 ± 0.015
40	8.75 ± 3.49	0.038 ± 0.014
50	11.83 ± 5.16	0.040 ± 0.016
60	13.22 ± 5.40	0.034 ± 0.013
70	12.63 ± 5.98	0.038 ± 0.013
80	12.51 ± 5.09	0.035 ± 0.010
90	12.85 ± 7.45	0.035 ± 0.012
100	7.55 ± 7.33	0.035 ± 0.012
200	8.36 ± 9.27	0.034 ± 0.012
300	6.01 ± 5.78	0.035 ± 0.013
400	3.49 ± 3.42	0.030 ± 0.016
500	3.19 ± 3.89	0.028 ± 0.014
1000	2.51 ± 2.65	0.030 ± 0.015
2000	2.23 ± 3.18	0.029 ± 0.015
5000	1.37 ± 2.82	0.030 ± 0.014
10,000	2.18 ± 2.24	0.027 ± 0.012

## Data Availability

Not applicable.
